# Survey of European pet owners quantifying endoparasitic infection risk and implications for deworming recommendations

**DOI:** 10.1186/s13071-018-3149-1

**Published:** 2018-11-01

**Authors:** Jessica McNamara, Jason Drake, Scott Wiseman, Ian Wright

**Affiliations:** 1 Elanco Animal Health, Lilly House, Priestley Road, Basingstoke, Hampshire RG24 9NL UK; 2 Elanco Animal Health, 2500 Innovation Way, Greenfield, IN 46140 USA; 3 Mount Veterinary Practice, 1 Harris St, Fleetwood, FY7 6QX UK; 4 ESCCAP UK & Ireland, PO Box 358, Malvern, WR14 9HQ UK

**Keywords:** ESSCAP, Risk assessment, Endoparasites, Deworming, Zoonosis, *Toxocara*, Parasite control, Dogs, Cats

## Abstract

**Background:**

Zoonotic endoparasites pose risks to pets and people. The European Scientific Counsel Companion Animal Parasites (ESCCAP) created risk groupings for dogs (A-D) and for cats (A-B), with the highest risk pets (Group D dogs and Group B cats) receiving the most frequent testing and/or deworming recommendations. Little information exists on current deworming behaviours across Europe, alignment to accepted guidelines and the percentage of dogs and cats falling into ESCCAP groups. The study objectives were to evaluate the reported infection-risk behaviours of dogs and cats and assesses whether deworming frequency reported by pet owners complied with recommended deworming frequencies.

**Methods:**

A total of 5001 pet owners from five different countries (France, Germany, Spain, Sweden and the UK) were surveyed regarding endoparasite infection risk and the frequency of deworming of dogs and cats. For the purposes of this study, ESCCAP risk groups for cats (A-B) were converted into four risk groups (A-D) using the additional risk factors outlined in the ESCCAP guidelines. This allowed direct comparison between cats and dogs as well as grouped higher risk cats into the appropriate deworming frequency.

**Results:**

The three most common risk factors identified for dogs were contact with: other dogs, snails or prey; children or the elderly; going off lead outside their own garden. 85–98% of all dogs had risks putting them into Group D, the highest risk group. The three most common risk factors identified for cats were: hunting; catching prey; contact with children or the elderly. Using these revised groups, 33–68% of cats were in Group D. Despite the majority of dogs and cats falling into a risk category where ESCCAP recommends monthly deworming, dogs and cats averaged 2.3 and 2.2 dewormings per year, respectively. This frequency was less than the four times a year dosing frequency demonstrated to be required to reduce zoonotic *Toxocara* spp. ova shedding.

**Conclusions:**

Overall, 93% of dogs and 54% of cats fell into Group D, the highest risk group. Deworming frequencies were considerably less than recommended by ESCCAP or required to both reduce zoonotic risk and improve pet health. Improved treatment compliance is needed.

**Electronic supplementary material:**

The online version of this article (10.1186/s13071-018-3149-1) contains supplementary material, which is available to authorized users.

## Background

There are a number of potentially zoonotic endoparasites found in dogs and cats that pose a risk to either pet health or public health including, but not limited to, *Toxocara* spp., *Echinococcus* spp., *Taenia* spp., *Dipylidium caninum*, *Dirofilaria* spp. and *Thelazia callipaeda* [1–3]. A good understanding of the epidemiology and risk factors associated with infection is required to ensure correct deworming procedures are applied to protect dogs and cats [1]. Several studies have identified these risk factors, and these have been summarised in the most recent European Scientific Counsel Companion Animal Parasites (ESCCAP) guidelines [2].

ESCCAP provides research-based independent advice regarding risk factors and recommended deworming frequency. In particular scope to this study are their guidelines ‘Worm Control in Dogs and Cats’ [2] which aims to deliver information for veterinarians and owners to more effectively control endoparasite infections in dogs and cats and reduce the zoonotic potential of certain parasites. A treatment regime designed specifically for each pet, based on individual assessment of risk factors should improve treatment efficiency [1].

In the literature there is evidence that dogs and cats are dewormed for a combination of pet health and public health reasons [4, 5]. Despite the risks from zoonotic endoparasites in dogs and cats, previous studies report in existing scientific literature that compliance to veterinary and guideline advice is poor [4–6]. The historical evidence points towards the fact that the current knowledge of pet owners is insufficient to expect them to make sound decisions on routine deworming [4–7].

It is accepted that an increase in pet travel along with certain climatic changes can influence the epidemiological situation of certain endoparasites, meaning that targeted and risk based worming have become even more important in recent times [2, 8]. Routine treatment and prevention of endoparasites depends on legislation in individual countries and information available to veterinary professionals including parasite epidemiology, owner education and individual risk assessments. It is advised within the ESCCAP guidelines that any deworming protocol should be on the advice of a veterinarian [2].

There is a paucity of information on whether current deworming behaviour across Europe is aligned to accepted guidelines. There is also a lack of information on the relative relevance of specific risk factors to individual animals. ESCCAP guidelines have been published to aid veterinarians to assess individual animal risk and prescribe accordingly, but the ability of owners and vets to assess risk accordingly and to comply with the recommended deworming frequency is unknown. The percentage of dogs and cats falling into each of the different ESCCAP risk categories is also unknown.

There are several studies that have been published looking at specific parasite exposure risks in specific countries or regions, but none have surveyed a larger population of cat and dog owners across several countries [6]. Most studies looking at deworming protocols and risk factors for pet infection and/or human exposure are specific to a geographical location, age group of dogs or cats or looked at faecal samples for positive diagnosis and subsequent identification of risk factors [9–13]. These include a study that was carried out in Canada to determine current recommended small animal deworming protocols and to compare these protocols with established guidelines, but focused mainly on puppy and kitten protocols and less on routine deworming of adult animals [14]. The study concluded that increased education of vets regarding the need for deworming was warranted.

Particular risk factors for endoparasite infection highlighted in the ESCCAP guide include freedom to roam, contact with dogs/cats outside the resident household, eating carrion or faeces of conspecifics or eating prey. Additional considerations include the age of the animal (e.g. puppies need to be dewormed more frequently), pregnancy/lactation status, eating slugs/snails, contact with children or immunocompromised individuals (increased zoonotic risk) and travel to certain areas (e.g. areas with endemic *Echinococcus* spp.). ESCCAP have published a flowchart as part of these guidelines, for both dogs and cats that takes these risk factors into consideration and forms a guideline for veterinarians to help them define individual risk and thus recommended deworming frequency [2].

The primary objective of this study was to evaluate the reported infection and transmission risks of dogs and cats based on owner observed and reported behaviours, interpreted with the most recent ESCCAP guidelines. As part of this, data were collected regarding which risk factors, identified within the ESCCAP guidelines, were present for individual animals. Additionally, we assessed whether current owner-reported deworming frequencies comply with the recommended deworming frequency laid out within the guidelines.

## Methods

An online survey was conducted among cat and dog owners in five major European countries: France, Germany, Spain, Sweden and the UK. These countries were identified as some of the most important EU deworming markets where investigation of deworming habits amongst pet owners would provide key insights into pet infection and transmission risks and pet owner behaviors. The survey ran from the 3rd July 2017 to the 14th July 2017.

### Key research questions

The survey was conducted in order to answer the following questions:

- What share of dogs and cats has a specific lifestyle/ risk of catching worms or transmitting zoonotic helminths?
- How often are dogs and cats dewormed with regard to their lifestyle?
- How often should dogs and cats be dewormed with regard to their lifestyle and geographical location?

### Design, setting, sample

The target group was defined as cat and dog owners who own at least one cat and/or dog and were responsible for the pet’s health care, product purchase and veterinarian visits. The target group selection criteria included cat and dog owners who: (i) are at least 18 years of age; (ii) own at least one cat and/or dog but less than 10; (iii) are responsible for the cat’s/ dog’s health care, product purchase and veterinarian visits; (iv) visit the veterinarian with the cat or dog at least once a year (except Sweden); (v) do not breed or trade cats and/or dogs for professional reasons.

Target respondents were recruited *via* a custom online panel of cat and dog owners. In order to achieve a target sample of *n* = 500 dog owners and *n* = 500 cat owners per country, a total number of 128,545 people, pre-screened for their ownership of a cat and/or dog, were sent an invitation email with a link to the survey in the respective country language which was centrally hosted on a secure server.

The survey was introduced as confidential and anonymous market research and potential respondents were assured that any information they provided would be combined with the responses of other respondents. Respondents were offered a small incentive of 2.10 € for completing the questionnaire in order to increase the response rate. The survey system stopped accepting responses after the quota of 500 cat owner and 500 dog owner responses per country was met. The exception was with dog owners in Spain, where two surveys were underway at the same time when the maximum submissions was reached, resulting in 501 dog surveys instead of 500.

At the beginning of the survey, several screening questions ensured participants could be included in the specific target group. Participants not meeting the criteria for inclusion in the survey were not allowed to complete the survey and were captured as “screen out”.

To be eligible for participation in the survey, respondents had to be at least 18 years-old and own at least one cat and/or dog. If both dogs and cats lived in the household, participants were randomly assigned to either the cat or the dog group. Cat and dog owners were not allowed to participate if they had more than 10 cats or dogs as this might not be typical households and could bias the results. Moreover, breeders and traders were also excluded from the survey as they might be more aware of the pets’ risk of catching worms and might show a different deworming routine which could bias the results. To personalize the questionnaire for the respondents, they were asked to enter the name of the cat or dog they wanted to talk about in the following questions. Furthermore, cat and dog owners had to, as a minimum, share the responsibility for the cat or dog with other members of their household or to have sole responsibility for the cat’s or dog’s health, product purchase and veterinarian visits. Except for Sweden, cat and dog owners also had to visit the veterinarian at least once a year to meet the target group criteria of well-cared dogs and cats. Sweden was included in the study to assess if different attitudes to deworming in Scandinavia and different legal requirements for veterinary prescribing significantly affected deworming frequency. In Sweden, veterinary clinics are not allowed to sell medical products except in acute, life-saving situations or for in-clinic treatments. Small pack size dewormers and some ectoparasiticides are sold over the counter (OTC) without a prescription from pharmacies exclusively, with large packs requiring a prescription. This meant that for Sweden to be included, it was not appropriate to include visiting the veterinarian once a year as a stipulation for completing the study.

In order to ensure a representative sample of the target group, quotas were set according to latest demographic statistics about cat and dog owners in the respective countries. The quotas related to age and gender, region, size of household (incl. number of children in the household) and employment status. The demographic data were acquired from a variety of public reports [15–24]. Additional demographic data were acquired from the proprietary “Pet Owner Survey 3 - March 2013”, a multi-client survey developed in cooperation between several Animal Health Companies within Centre Europeen d’Etudes Pour La Sante Animale (CEESA), Brussels, Belgium.

The main survey contained 9 or 10 questions (see Tables 1 and 2 for the dog and cat surveys, respectively). First, cat and dog owners reported how often their cat or dog is dewormed within a year. This question was asked first to ensure that the results were not biased after cat and dog owners read other statements about, e.g. deworming products. Next, questions about the cats’ and dogs’ lifestyle followed.

**Table 1:** Survey questions for dog owners

Question number	Question	Answer options
1	Which country do you live in?	Free text response
2	What is your post code?	Free text response
3	Is your dog less than 6 months old?	Yes/No
4	Is your dog supervised and exercised only in your garden? (no contact with public places, other dogs, slugs/ snails, raw meat or prey animals)	Yes/No
5	Does your dog ever catch animals (or pick up carcasses) such as rabbits or mice?	Yes/No
6	Does your dog live with or visit children or the elderly?	Yes/No
7	Does your dog exercise off the lead?	Yes/No
8	Does your dog eat any raw meat?	Yes/No
9	Does your dog eat slugs, snails, grass or dig in the garden?	Yes/No
10	How often do you currently deworm your dog?	1–2× per year/4× per year/4+ × per year/Monthly/Never or rarely

**Table 2:** Survey questions for cat owner

Question number	Question	Answer options
1	Which country do you live in?	Free text response
2	What is your post code?	Free text response
3	Is your cat less than 6 months old?	Yes/No
4	Is your adult cat kept indoors all the time (And does not eat raw meat)?	Yes/No
5	Does your cat go outdoors but not hunt or eat raw meat?	Yes/No
6	Does your cat ever catch prey such as mice and birds?	Yes/No
7	Does your cat live with or visit children or the elderly?	Yes/No
8	Does your cat eat raw meat?	Yes/No
9	Does your cat eat slugs, snails, grass or dig in the garden?	Yes/No
10	How often do you currently deworm your cat?	1–2× per year/4× per year/4+ × per year/Monthly/ Never or rarely

As previously discussed, the current ESCCAP guidelines are widely recognised as the industry standard for guidance on recommended deworming frequency in dogs and cats [2]. In these, there are certain risk factors that are highlighted as having a direct impact on recommended deworming frequency. A risk assessment questionnaire was designed based on these guidelines, along with specific interpretation guidelines that lead to a recommended deworming frequency based on responses given. For each country ESCCAP guidelines utilized [2]. For Germany and the UK, local ESCCAP materials were also utilised [25, 26]. The exception to this was Sweden. The same survey was used for Sweden, but interpretation was based on local key opinion leader input, University guidelines and government guidelines. This is because, while the ESCCAP guidelines are designed to guide veterinary decision making Europe wide, it is also clearly specified in the guidelines that any final decision on parasite prevention measures should be made by the veterinary surgeon on the basis of individual risk assessment and within the legal framework of the individual country. The legal framework in Sweden is very different from that of the other countries surveyed as veterinary clinics are not allowed to sell medical products except in acute, life-saving situations or for in-clinic treatments. Routine prescription preventative treatments are also not advised unless there is confirmation of infection by diagnostic testing or there is exceptional local risk. The use of local key opinion leader output and legal advice is therefore rational for this study in the case of Sweden.

The questions were different for cat and dog owners and were developed based upon the European ESCCAP guidelines as well as ESCCAP guidelines locally adapted in each of the surveyed countries [2, 25, 26]. These questions covered, for example, the cats’ and dogs’ age, access to outdoors, and whether they catch and/or eat prey animals. Moreover, it was determined if the cat or dog lives with children or the elderly. In addition, cat and dog owners were asked about their opinion with regard to current deworming products. Thus, general prejudices and misunderstandings of cat and dog owners with regard to deworming products could be detected. To classify cat and dog owners into a specific pet owner segment, they were also asked about their relationship with their cat or dog as well as with their veterinarian. To learn more about where cat and dog owners’ seek information from, it was additionally asked to whom or which source of information they would refer for advice regarding deworming of their cat or dog.

The survey questions followed EU and individual country ESCCAP guidelines for assessing risk of parasite infection [2, 25, 26]. The pet owner responses regarding pet behavior and exposure risks place the pet into 4 distinct risk groups, as defined in Table 3 (dogs) and Table 4 (cats). Although ESCCAP guidelines only have 2 risk groups for cats (A and B), there are also risk factors outlined in the ESCCAP guidelines in a table labelled “additional treatments for cats” [2]. This table indicates the need for monthly deworming of cats in close contact with young children or immune suppressed individuals, and to consider 4–6 times a year deworming for cats not under close supervision. These and feeding of raw diets were used to formulate 2 further risk groups for cats (C and D). The “additional treatments for dogs” advises monthly deworming for dogs in close contact with young or immune compromised so these dogs were included in risk group D. In this way, the results could be reported consistently for cats and dogs, while acknowledging the conditions in the guidelines where monthly deworming is indicated for cats. An exception to this was made for Sweden, where kittens and purely indoor cats are each considered as separate groups. Swedish risk groups for cats were added to Groups 0–5 with 0 for kittens (146 cats) and 5 for purely indoor cats (9 cats). The 155 Swedish cats in Groups 0 and 5 were omitted from the analysis across all 5 countries. Swedish cats in Groups 1–4 were aligned to Groups A-D. This also allows Swedish data to be compared in a consistent way.

**Table 3:** Dog risk group definitions

Dog Risk Group	Description	EU ESCCAP recommended deworming frequency
A	Older than 6 months, lives indoors only or goes outdoors but has no direct contact with parks, sandpits, playgrounds, (faeces from) other dogs and cats, snails and slugs, raw meat or prey	1–2 times per year
B	Older than 6 months, goes outdoors and has direct contact with parks, sandpits, playgrounds, and (faeces from) other dogs and cats; but does not eat prey animals and/or snails and slugs and/or goes outdoors to hunt and does not eat raw meat	4 times per year
C	Older than 6 months, goes outdoors and has direct contact with parks, sandpits, playgrounds, and (faeces from) other dogs and cats and eats prey animals and/or snails and slugs and/or goes outdoors to hunt and eats raw meat	> 4 times per year
D	Is less than 6 month-old; *or* lives in a fox tapeworm (*Echinococcus multilocularis*) endemic area; *or* eats prey animals and/or goes outdoors to hunt; *or* lives indoors, eats raw meat and lives with children/elderly	Monthly

**Table 4:** Cat risk group definitions

Cat Risk Group	Description	EU ESCCAP recommended deworming frequency
A	Cat lives indoors. Infection pressure with worm stages is low, eating rodents unlikely	Treat 1–2 times per year against roundworms, or 1–2 times per year fecal exam and treatment according to findings
B	Cat is free to roam outdoors. Infection pressure with worm stages is high, eating rodents likely	Treat against roundworms and tapeworms at least 4 times a year
C^a^	Cat eats prey animals and/or goes outdoors to hunt and eats raw meat	Treat against roundworms and tapeworms more than 4 times per year
D^a^	Cat is free to roam outdoors and shares home with young children or immunocompromised individuals	Deworm once a month, or examine faecal samples once a month and treat according to findings

^a^ESCCAP Cat Risk Groups include A and B only. Additional risk factors in the ESCCAP guidelines were used to create Groups C and D for consistency in reporting and comparison of dog and cat results

While Table 3 and Table 4 show the general European ESCCAP risk assessment and deworming guidelines for dogs and cats, respectively, additional adaptations were made to align with each country’s local guidelines in effect at the time of the survey. The final survey deworming frequency recommendations are based upon the survey responses as outlined in Table 5 (dogs) and Table 6 (cats).

**Table 5:** Recommended canine deworming frequencies based upon country risk assessments

Dogs^a^	France	Germany	Spain	Sweden	UK
Puppy	Monthly	Monthly	Monthly	After 3 and 6 months	Monthly
Only exercised in garden, supervised	1–2× yearly	1–2× yearly	1–2× yearly	1–2× yearly	4× yearly
Catches animals	Monthly	Monthly	Monthly	4× yearly	Monthly
With children/elderly	Monthly	Monthly	Monthly	No change in deworming based upon this risk	Monthly
Allowed off-lead	> 4× yearly	> 4× yearly	> 4× yearly	4× yearly	> 4× yearly
Fed/eats raw meat	> 4× yearly	> 4× yearly	> 4× yearly	4× yearly	> 4× yearly
Eats slugs/snails	Monthly	Monthly	Monthly	4× yearly	Monthly

^a^If “Yes” in survey

**Table 6:** Recommended feline deworming frequencies based upon country risk assessments

Cats^a^	France	Germany	Spain	Sweden	UK
Kitten	Monthly	Monthly	Monthly	After 3 and 6 months	Monthly
Indoor	1–2× yearly	1–2× yearly	1–2× yearly	Never	4× yearly
Outdoor	4× yearly	More than 4× yearly	4× yearly	4× yearly	4× yearly
Eats prey	> 4× yearly	> 4× yearly	> 4× yearly	> 4× yearly	> 4× yearly
With children/ elderly	Monthly	Monthly	Monthly	No change in deworming based upon this risk	Monthly
Fed/eats raw meat	> 4× yearly	> 4× yearly	> 4× yearly	4× yearly	> 4× yearly

^a^If “Yes” in survey

### Statistical methods

The association between risk group and the frequency of deworming, adjusting for the effect of country, was investigated by constructing stratified contingency tables of frequency of deworming against risk groups and testing the null hypothesis of no association between the variables using Cochran-Mantel-Haenszel (CMH) test statistics and was performed using SAS v9.4. Owners reported that their pets were dewormed up to 20 times per year (dogs) or 25 times per year (cats). In order to minimize the number of categories with zero frequencies, all responses reporting more than 12 dewormings per year were grouped together as ‘> 12’ in the analysis. Results from the 155 owners in Sweden where cats were classified in risk groups 0 and 5 were excluded from analysis since these categories were not appropriate in the other countries.

The proportion of pets aligned to deworming recommendations, according to risk group was estimated according to Table 5 (dogs) and Table 6 (cats). It should be noted that the monthly recommendation does not necessarily mean that the animal must be dewormed 12 times. For example, a dog of 8 weeks of age would be recommended monthly dosing based on age, if this dog had no further risk factors it would be compliant if dewormed 6 times during the year - 4 times up to 6 months of age and then 1 or 2 times after that.

## Results

Surveys were completed by a total of 5001 pet owners, with 500 cat owners from each of the 5 countries surveyed and 500 dog owners from 4 of the 5 countries surveyed. In Spain, 501 dog owners completed the survey. In order to reach 5001 pet owner responses, a total number of 12,055 people followed the invitation and visited the entry page. Of these, 444 terminated prior to completing the survey. Moreover, 4943 did not match the target group and were screened out. 1667 were excluded from the survey due to quota outs. The average time required to complete the survey was 7.5 minutes (see Table 7 for completion details by country).

**Table 7:** Number of completed survey responses by country

Country	Total	France	Germany	Spain	Sweden	UK
Total invited	128,545	19,855	18,020	35,830	31,950	22,890
Non-response	116,490	17,871	16,216	32,657	29,168	20,578
Cancelled	444	71	35	145	100	93
Screen out^a^	4943	738	592	1408	1284	921
Quota out^b^	1667	175	177	619	398	298
Total completed	5001	1000	1000	1001	1000	1000
Dog owners	2501	500	500	501	500	500
Cat owners	2500	500	500	500	500	500

^a^Screen out: respondent did not meet selection criteria listed in Methods

^b^Quota out: respondent attempted to complete the questionnaire after participant limit reached

**Fig. 1:**
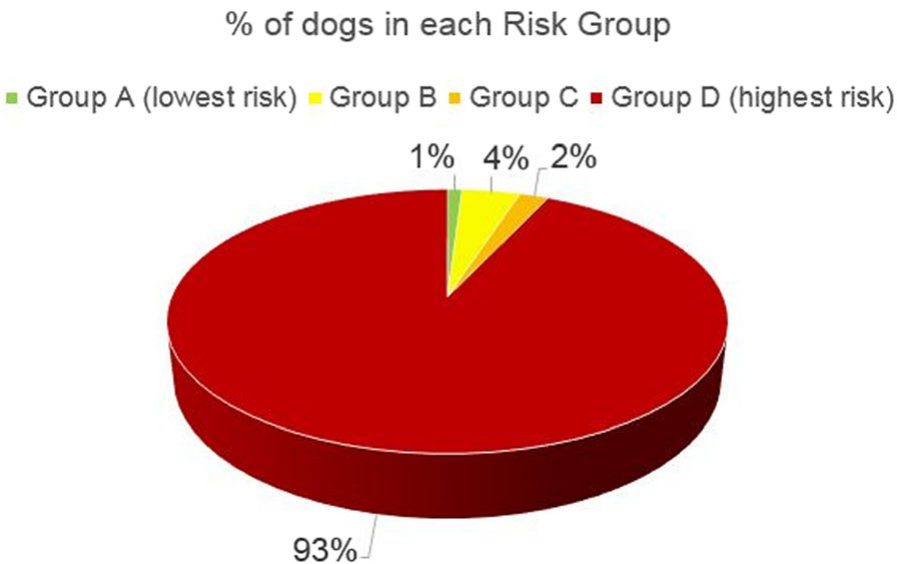
Percent of dogs in each ESCCAP risk group

Overall for dogs (*n* = 2501), 97% of dogs owned by survey participants were > 6 months of age. The three most common risk factors reported by owners related to either infection of their dogs or people around their dogs, starting with the most common, included contact with other dogs, snails or prey, contact with children or the elderly and going off lead outside their own garden (Table 8). Depending upon the country, 85–98% of all dogs had exposure risks putting them into Risk Group D, the highest risk group (Table 9, Fig. 1). Greater than 84% of dogs had contact with children or the elderly and greater than 84% of dogs had contact with other dogs, snails or prey. Interestingly, 51% of dogs reportedly ate snails, slugs, ate grass or dug in the garden. For dogs that went outside beyond their own garden, 54% were allowed off-lead and 16% had caught prey animals. Of the dogs that did not hunt, 19% were fed raw meat. Despite the majority of dogs ending up in Risk Group D, dogs received only 2.3 doses per year on average. ESCCAP recommends monthly deworming for dogs in Risk Group D. Full canine details, by country, are reported in Table 8.

**Table 8:** Canine questionnaire results

Country	France	Germany	Spain	Sweden	UK	Average
> 6 months of age (%)	97	97	96	98	98	97
Contact with children/elderly (%)	75	91	91	81	81	84
Garden only access (%)	39	22	20	16	11	22
If goes outside garden - off lead (%)	29	76	20	70	73	54
Contact with other dogs, snails or prey (%)	83	89	75	93	82	84
Eats slugs, snails, grass or digs in garden (%)	68	67	33	54	33	51
Catch prey animals (%)	22	19	14	14	13	16
Fed raw meat (of those that do not hunt) (%)	13	39	5	28	12	19
Dewormings per year	2.3	2.1	3.1	1	3.1	2.3

**Table 9:** Canine risk group results

Country	France	Germany	Spain	Sweden	UK	Average
Dogs (*n*)	500	500	501	500	500	
Risk group A (%)	2	1	1	1	0	1
Risk group B (%)	2	0	2	14	2	4
Risk group C (%)	7	1	0	0	1	2
Risk group D (%)	89	98	96	85	97	93

**Fig. 2:**
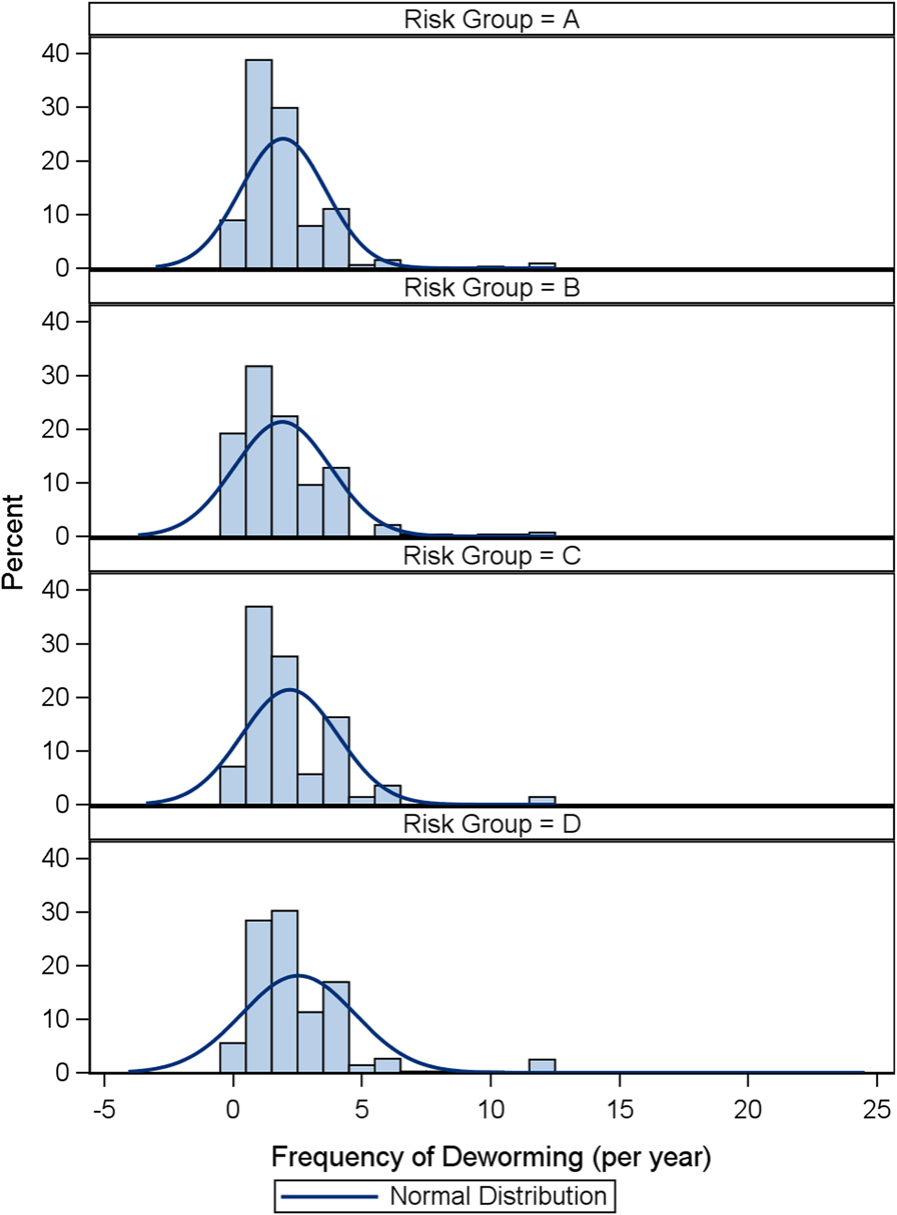
Canine distribution of frequency of deworming by risk group

In dogs, the mean frequency of deworming was 2.0, 1.4, 2.0 and 2.4 times per year for risk categories A, B, C and D, respectively (Additional file 1: Table S1). Similarly, the median frequency was 2, 1, 2, 2 times per year. These statistics illustrate that there was little or no location shift of the centre of the frequency distribution. However, the CMH test provided evidence of an association between frequency of deworming and risk category (*Q*_*CSMH*_ = 4.36, *df* = 1, *P* < 0.04). Closer investigation of the distributions (Fig. 2) revealed that this association was most likely driven by the longer tail in risk group D. That is, a small number of dogs in the highest risk group were dewormed more frequently. The mean frequency of deworming did not increase, but a small number of owners were recognizing the risks to their pets and were deworming more frequently. However, the proportion of pets in risk group D dewormed at least 6 times (in alignment with ESCCAP recommendations of monthly deworming) was only 4.7%. The vast majority of dogs in this survey were in risk group D (97%) so this result indicates that only a small number of dogs were being dewormed according to recommendations. Even this small proportion may be an overestimate of the dogs dewormed in alignment with recommendations since a frequency of deworming of 6 times a year was considered sufficient for alignment with a recommendation of monthly dosing. There were some notable differences in deworming frequency between the different countries, particularly in dogs in the highest risk category where there were 3 distinct levels. Owners of the highest risk category dogs in Spain and UK dewormed their pets approximately 3 times a year, those in France and Germany approximately twice a year and in Sweden only once per year.

**Fig. 3:**
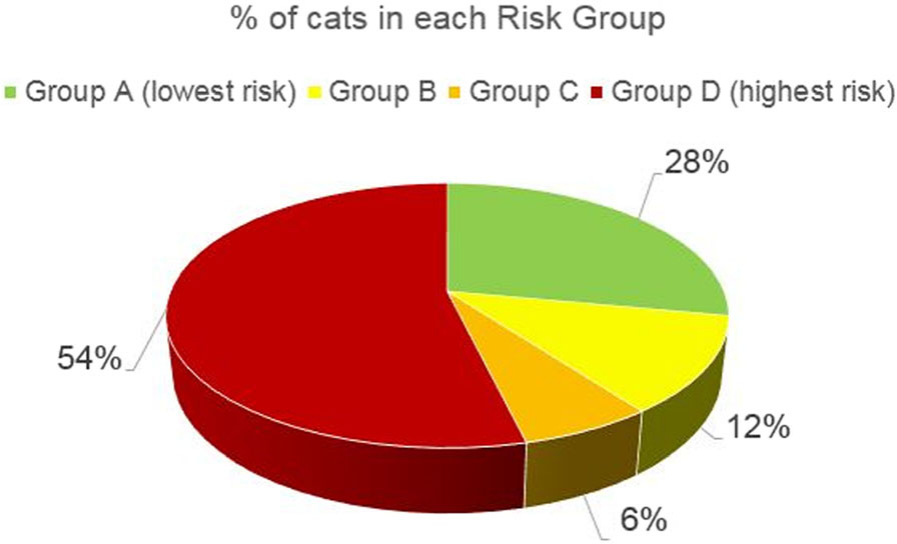
Percent of cats in each ESCCAP risk group

Overall for cats (*n* = 2500), 96% of cats owned by survey participants were > 6 months of age. The three most common risk factors reported by owners, related to either infection of their cats or people around their cats, starting with the most common, included hunting, catching prey and contact with children or the elderly (Table 10). Depending upon the country, 33–68% of cats ended up in Risk Group D based upon the exposure risks reported by their owners (Table 11, Fig. 3). Interestingly, 65% of cats had contact with children or the elderly. 77% of cats hunted, 73% caught prey and 16% of the cats that did not catch prey or go outside were fed raw meat. Despite 50% of cats in Risk Group D, cats received only 2.2 dewormings per year on average. ESCCAP recommends monthly deworming for cats in Risk Group D. Recommendations for cats were slightly different in Sweden from the ESCCAP guidelines. Kittens and purely indoor cats were put in separate risk groups (0 and 5), with recommendations that kittens be dewormed at 3 and 6 months, and purely indoor cats not be dewormed. Out of the 500 cats surveyed in Sweden, 155 were in Groups 0 and 5. Therefore, deworming totals for cats in Sweden, as shown in Table 11, added up to 69%, with 31% of the Swedish felines categorized as either kitten or purely indoor cats. Additional feline details, by country, are reported in Table 10.

**Table 10:** Feline questionnaire results

Country	France	Germany	Spain	Sweden	UK	Average
> 6 months of age (%)	97	99	91	95	98	96
Contact with children/elderly (%)	57	67	79	71	52	65
Kept only indoors (%)	34	50	71	39	20	43
If goes outside garden - hunt (%)	87	88	63	74	72	77
If goes outside garden - catch prey (%)	80	85	64	73	62	73
Fed raw meat (of those that do not go outside or catch prey) (%)	10	32	6	23	8	16
Dewormings per year	2.3	1.7	2.6	1.2	3.1	2.2

**Table 11:** Feline risk group results

Country	France	Germany	Spain	Sweden^b^	UK	Average
Cats (*n*)	500	500	500	500	500	
Risk group A (%)	33	34	62	3	0	26
Risk group B (%)	3	3	3	18	29	11
Risk group C^a^ (%)	11	12	2	0	3	6
Risk group D^a^ (%)	53	50	33	48	68	50

^a^ESCCAP Cat Risk Groups include A and B only. Additional risk factors in the ESCCAP guidelines were used to create Groups C and D for consistency in reporting and comparison of dog and cat results

^b^155 (31%) of cats in Sweden were categorized as kittens or purely indoor cats and are not presented in this table

Interestingly, while 93% of dogs were in Risk Group D, 50% of cats were in Risk Group D. 37% of cats were in either Risk Group A or Risk Group B while, with the exception of Sweden, only 0–2% of dogs were in Risk Groups A or B. In Sweden, 15% of dogs were in Risk Groups A or B. ESCCAP recommends deworming Risk Groups A and B either 1–2 times per year or 4 times per year, respectively. A higher percentage of dogs than cats have contact with children or the elderly. While cats were more likely to hunt or catch prey, dogs were somewhat more likely to be fed raw meat.

The mean frequency of deworming in cats was 1.9, 1.9, 2.2 and 2.5 times per year for risk categories A, B, C and D, respectively (Additional file 1: Table S2). Similarly, the median frequency was 2, 1, 2 and 2 times per year. This pattern is similar to that observed for dogs and the statistics again illustrate that the centre of the frequency distribution did not clearly increase with risk. However, for cats there was more indication of a location shift than for dogs, for example the mean frequency of deworming was 2.5 in the highest risk group compared to 1.9 in the lower risk groups. The CMH test in cats again provided evidence of an association between frequency of deworming and risk category (*Q*_*CSMH*_ = 61.86, *df* = 1, *P* < 0.0001) and was more pronounced than observed in dogs.

**Fig. 4:**
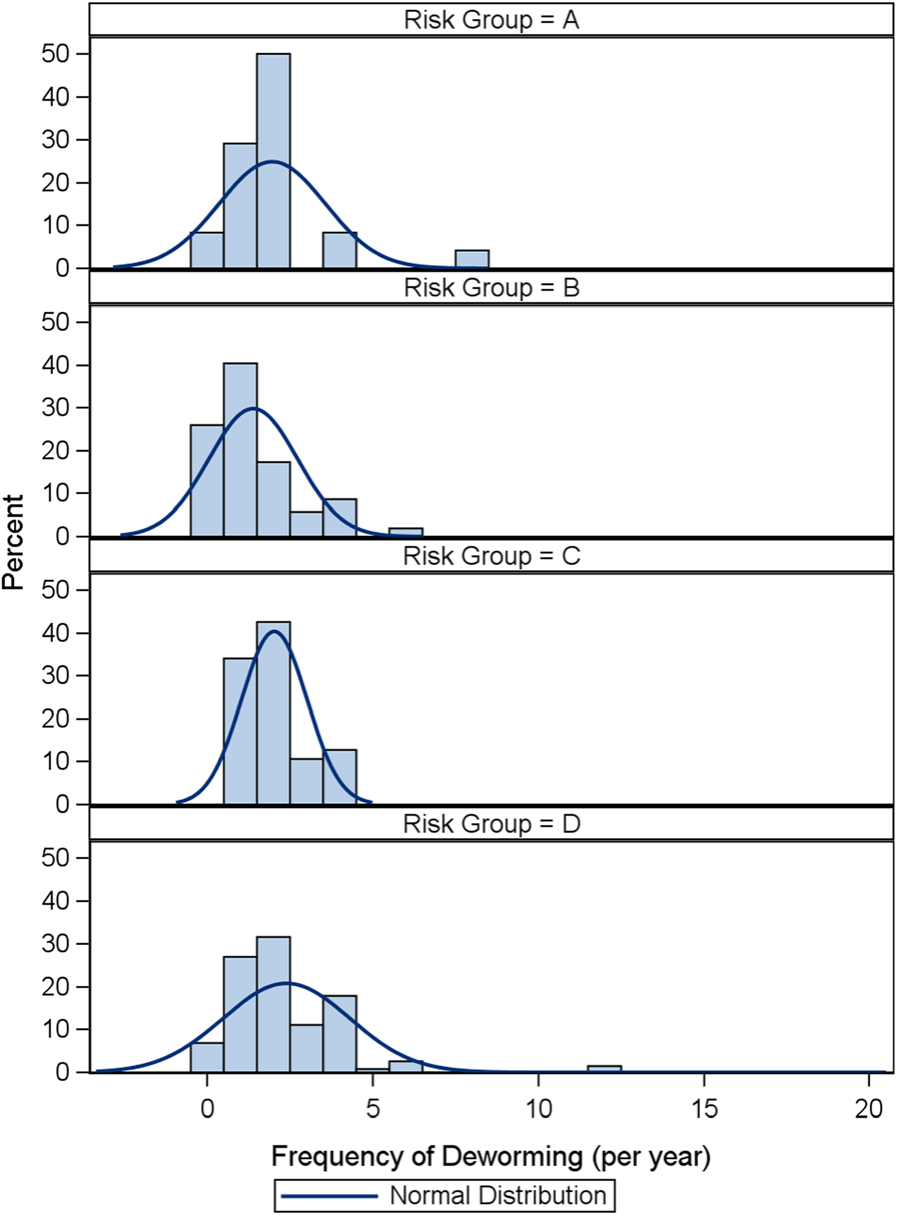
Feline distribution of frequency of deworming by risk group

There was evidence of longer tails in the distribution in the higher risk groups but this was not as pronounced as for dogs (Fig. 4). However, the findings are similar; a small number of cats in the higher risk groups were dewormed more frequently. The proportion of pets in risk group D dewormed at least 6 times (in alignment with ESCCAP recommendations of monthly deworming) was 6.1%.

## Discussion

To the authors’ knowledge, this is the first large scale objective study of deworming treatment behaviour among cat and dog owners. It is also the first to assess risk factors that would affect deworming frequency based on owner information from across Europe. Information derived from studies such as this one is important, as preventative treatment protocols based on risk assessment are only as effective as how accurately pet owners put them into practice.

The results of this survey indicate that a significant majority of dogs across Europe (93%) fall into the category D of the ESCCAP treatment recommendations for deworming frequency and yet in no country was the monthly recommended treatment frequency in this group met (Table 8). The high percentage of dogs in high-risk Group D should be treated with caution as the respondents to the questionnaire were not randomly selected and there may be response bias which is artificially affecting the results, e.g. owners more likely to respond to questionnaires may be in age groups more likely to have children, live in rural areas allowing their dogs to roam further etc. Respondents to surveys, however, may also be more involved and interested in pet care and be more likely to deworm and/or follow vet or ESCCAP advice. It is likely therefore, on the basis of these results, that many dogs are at increased risk of parasitic worm infection and/or being in contact with groups at high risk of zoonotic infection.

Although a lower proportion of cats were in high risk Group D (50%), none of these cats were treated for worms at the recommended monthly treatment frequency (Table 9). With the exception of the UK (68%) and Spain (33%) as outliers, the proportion of cats in Group D was consistent across Europe. The differences in Spain and the UK may be due to differing trends in lifestyle, geography and the socioeconomic groupings of cat owners in these countries. Pet healthcare plans for example are popular in the UK. These require practices to consider deworming frequencies and encourage owners to put them into practice by paying for them on a monthly basis as part of an overall health plan. For increased uptake of these plans to be successful owners need to be made aware of the value of evidence based routine deworming practices and therefore, compliance increased. This cannot be concluded conclusively however, from the data in this study and warrants further investigation.

The higher proportion of cats in Groups A and B when compared to dogs is likely due to the relative ease with which cats and be kept indoors. The figures from this study, also suggest that they are less likely to contact children which may be due to the inherent behavioural differences between dogs and cats.

A smaller but significant proportion of cats were fed raw diets. This is a growing trend across Europe [27] which has the potential to expose dogs and cats to parasitic worm infection. Commercial processed raw diets will have undergone meat inspection to human food standards and will have been frozen to -18 °C for at least 7 days to kill potential parasitic life stages. The potential for home prepped raw diets to be fed, however, as well as for dogs and cats to be fed raw offal and meat from a number of sources that have not undergone meat inspection, means that this route needs to be considered in deworming frequency. None of the dogs and cats being fed raw meat in this study, on average, were on an effective deworming frequency.

There is no evidence that deworming frequencies of less than four times a year in dogs and cats has any impact on reducing *Toxocara* spp. egg shedding in the faeces, and therefore zoonotic risk [13, 28]. This is of particular concern as only 2% of UK dogs and no other dogs in this survey across Europe on average were treated at or above this frequency (Table 12). Similarly, no cats in the survey met this minimum treatment requirement to reduce shedding (Table 13). Given that 65% of cats and 84% of dogs had contact with children and the elderly, and that children are at particular risk of toxocarosis [1] this failure to treat adequately represents a potentially significant and likely underestimated health risk. Given that seropositivity in human populations across Europe vary widely and that the proportion of dogs and cats in contact with children and the elderly were broadly the same [1], it can be concluded that other risk factors for toxocarosis are having a significant effect on human exposure. Nonetheless, there is an opportunity to reduce exposure risk by increasing deworming frequencies in high risk groups across Europe. *Toxocara* ova passed in faeces are not immediately infective and although it has been demonstrated that *Toxocara* spp. egg can embroyonate in the coats of dogs, it is not at as higher rate as in soil [29, 30]. *Toxocara* eggs however, are long lived in the environment and numbers of infective ova will increase in the environment if effective treatment of shedding dogs and cats with anthelmintic is not achieved. In the Netherlands, household dogs older than 6 months of age accounted for 39% of the overall *Toxocara* egg output. Intervention scenarios revealed that high compliance (90%) to the four times a year deworming advice would reduce the dog’s contribution from 39 to 28%. Alternatively, when 50% of owners would always remove their dogs’ faeces, dogs’ contribution would drop to 20% [31].

**Table 12:** Canine lifestyle and deworming frequency

Reported risk or behavior	France (*n* = 500)		Germany (*n* = 500)		Spain (*n* = 501)		Sweden (*n* = 500)		UK (*n* = 500)		EU Average (*n* = 2501)		ESCCAP recommended deworming frequency
	%^a^	*N* ^b^	%^a^	*N* ^b^	%^a^	*N* ^b^	%^a^	*N* ^b^	%^a^	*N* ^b^	%^a^	*N* ^b^	
Goes outside beyond garden, contact with other animals, eats slugs, snails, grass or raw meat and lives with children	21	2.5	11	2.4	18	3.5	9	0.9	7	3.7	13	2.62	Monthly
Goes outside only in garden, eats slugs, snails, grass or prey and lives with children	16	2.1	10	2.1	7	2.2	6	1.1	2	4.0	8	2.29	Monthly
Goes outside beyond garden off lead, eats slugs, snails, grass or raw meat and lives with children	12	2.4	43	2.1	8	3.0	35	1.1	25	3.5	25	2.42	Monthly

^a^Percent of dogs with behaviour or risk factor described

^b^Reported number of deworming treatments per year

**Table 13:** Feline lifestyle and deworming frequency

Reported risk or behavior	France (*n* = 500)		Germany (*n* = 500)		Spain (*n* = 500)		Sweden (*n* = 500)		UK (*n* = 500)		EU Average (*n* = 2500)		ESCCAP recommended deworming frequency
	%^a^	*N* ^b^	%^a^	*N* ^b^	%^a^	*N* ^b^	%^a^	*N* ^b^	%^a^	*N* ^b^	%^a^	*N* ^b^	
Goes outdoors, hunts/catches prey and lives with children	31	2.3	32	2.2	16	2.7	33	1.8	29	3.4	28	2.49	More than 4 times per year
Goes outdoors, hunts/catches prey	23	2.4	11	2.3	3	2.4	11	1.7	27	3.2	15	2.39	More than 4 times per year
Pure indoor cat	15	1.9	14	1.0	15	2.3	11	0.3	10	2.4	13	1.59	Never

^a^Percent of dogs with behaviour or risk factor described

^b^Reported number of deworming treatments per year

The health of dogs and cats will also be affected by inadequate deworming programs, especially in worms capable of causing potentially life-threatening conditions such as *Angiostrongylus vasorum* and *Dirofilaria immitis*. This study would suggest that many dogs and cats in countries endemic for heartworm are left unprotected. Owners surveyed however, may not recognize their heartworm preventative treatments as routine “deworming” and count long acting treatments such as moxidectin injections as single treatments. Regions where heartworm exposure is seasonal may also lead to monthly treatments for only part of the year, leading to a skewed average dosing frequency overall. These factors require further investigation to establish how many dogs and cats are being left exposed to heartworm infection but with such a low dosing average it can be concluded that a significant number of dogs and cats are being left unprotected. Ensuring adequate deworming frequency is achieved after accurate risk assessment is therefore important to reduce zoonotic risk and improve animal health.

Evidence is lacking as to what point implementation of adequate deworming regimes is failing. For appropriate deworming frequencies to be implemented veterinary professionals must be convinced of the health benefits to pets and to the public. They must also have access to current disease and lifestyle risk data to accurately advise treatment frequency. The advice must be presented to clients in a way that they understand both how to implement effective treatment and appreciate the value and importance of implementing them. Finally, owners must then remember to give treatments at the correct time and frequency.

Failure in any of these steps will reduce treatment frequency and further research should be targeted at identifying which steps are currently not working to ensure adequate deworming frequency. Until this information becomes available, the importance of conducting risk assessments for all dog and cat patients and prescribing appropriate deworming based upon ESCCAP guidelines needs to be emphasized to veterinary professionals and carried out. Effective methods of improving treatment compliance among pet owners such as apps, effective use of websites and social media as well as practice care plans should also be investigated and promoted.

## Conclusions

This study begins to address the shortage in Europe wide data regarding the lifestyles of pet dogs and cats in relation to their routine deworming requirements and actual treatment frequencies administered by owners. The large proportion of both dogs and cats in the highest risk lifestyle groups suggests that the majority of dogs and cats should be on a monthly deworming regime. In addition, to reduce zoonotic risk from *Toxocara* spp. infection, pets at any risk of significant *Toxocara* ova shedding or prolonged contact with children or immune suppressed individuals at increased risk of toxocarosis, should be dewormed at least 4 times a year. That only 2% of dogs and no cats in this study were being dewormed four times a year or more is therefore of major concern. Further research is required to establish if these trends in inadequate deworming frequencies are genuine, especially in high risk groups. Research is also required to establish where failure in communication and application of adequate deworming frequencies is occurring. Meanwhile, easy access to the latest data for Veterinary professionals is vital to ensure they can calculate risk accurately and give effective advice to clients. Veterinary professionals and the public both need to be engaged to impress the importance of adequate deworming regimes and aids given to pet owners to help them remember when and how these should be administered. Veterinary professionals have a responsibility to conduct risk assessments for all dog and cat patients and prescribe appropriate deworming based upon evidence based guidelines such as those produced by ESCCAP. Only by Veterinary professionals engaging with the public and giving accurate evidence based advice in an accessible way, will compliance be increased and both animal health increased, and zoonotic risk decreased as a result.

## Additional file

Additional file 1: **Table S1.** Canine frequency of deworming and alignment with ESCCAP recommendations. **Table S2.** Feline frequency of deworming and alignment with ESCCAP recommendations. (DOCX 17 kb)

## Abbreviations

ESCCAP: European Scientific Counsel Companion Animal Parasites
