# Supplementary material for: Survey of European pet owners quantifying endoparasitic infection risk and implications for deworming recommendations
Source: Parasit Vectors. 2018 Nov 1;11:571. doi: 10.1186/s13071-018-3149-1 (PMC6211546; doi:10.1186/s13071-018-3149-1)
Supplement: Supplementary file 1 — Table S1. Canine frequency of deworming and alignment with ESCCAP recommendations. Table S2. Feline frequency of deworming and alignment with ESCCAP recommendations. (DOCX 17 kb) [file 13071_2018_3149_MOESM1_ESM.docx]

**Additional file 1**

**Table S1** Canine frequency of deworming and alignment with ESCCAP recommendations

| Risk Group | Statistic | France  (n=500) | Germany  (n=500) | Spain  (n=501) | Sweden  (n=500) | UK  (n=500) | Overall  (n=2501) |
| --- | --- | --- | --- | --- | --- | --- | --- |
| A | Mean | 2.7 | 1.7 | 1.3 | 1.4 | - | 2.0 |
|  | Min | 1 | 1 | 1 | 0 | - | 0 |
|  | Q1 | 2 | 1 | 1 | 0 | - | 1 |
|  | Median | 2 | 2 | 1 | 1 | - | 2 |
|  | Q3 | 2 | 2 | 2 | 2 | - | 2 |
|  | Max | 8 | 2 | 2 | 4 | - | 8 |
|  | % aligned | 100% | 100% | 100% | 60.0% | - | 91.7% |
| B | Mean | 2.7 | 2.0 | 3.1 | 0.9 | 2.0 | 1.4 |
|  | Min | 1 | 2 | 1 | 0 | 1 | 0 |
|  | Q1 | 2 | 2 | 2 | 0 | 1 | 0 |
|  | Median | 2 | 2 | 3 | 1 | 1.5 | 1 |
|  | Q3 | 4 | 2 | 4 | 1 | 3 | 2 |
|  | Max | 6 | 2 | 6 | 4 | 4 | 6 |
|  | % aligned | 36.3% | 0.0% | 72.7% | 4.1% | 25.0% | 16.3% |
| C | Mean | 1.8 | 1.5 | 2.5 | - | 3.5 | 2.0 |
|  | Min | 2 | 1 | 2 | - | 2 | 1 |
|  | Q1 | 1 | 1 | 2 | - | 3 | 1 |
|  | Median | 2 | 1 | 2.5 | - | 4 | 2 |
|  | Q3 | 2 | 2 | 3 | - | 4 | 2 |
|  | Max | 4 | 3 | 3 | - | 4 | 4 |
|  | % aligned | 0.0% | 0.0% | 0.0% | - | 0.0% | 0.0% |
| D | Mean | 2.3 | 2.1 | 3.0 | 1.1 | 3.2 | 2.4 |
|  | Min | 0 | 0 | 0 | 0 | 0 | 0 |
|  | Q1 | 1 | 1 | 2 | 0 | 2 | 1 |
|  | Median | 2 | 2 | 3 | 1 | 3 | 2 |
|  | Q3 | 3 | 3 | 4 | 1 | 4 | 3 |
|  | Max | 12 | 12 | 20 | 12 | 12 | 20 |
|  | % aligned | 3.6% | 1.6% | 8.5% | 0.7% | 8.6% | 4.7% |

**Table S2** Feline frequency of deworming and alignment with ESCCAP recommendations

| Risk Group |  | France  (n=500) | Germany  (n=500) | Spain  (n=500) | Sweden  (n=345) | UK  (n=500) | Overall  (n=2345) |
| --- | --- | --- | --- | --- | --- | --- | --- |
| A | Mean | 1.9 | 1.2 | 2.4 | 0.4 | - | 1.9 |
|  | Min | 0 | 0 | 0 | 0 | - | 0 |
|  | Q1 | 1 | 1 | 1 | 0 | - | 1 |
|  | Median | 2 | 1 | 2 | 0 | - | 2 |
|  | Q3 | 2 | 2 | 3 | 1 | - | 2 |
|  | Max | 10 | 4 | 12 | 2 | - | 12 |
|  | % aligned | 93.9% | 84.8% | 95.5% | 35.7% | - | 91.1% |
| B | Mean | 1.5 | 1.7 | 2.4 | 1.1 | 2.4 | 1.9 |
|  | Min | 0 | 1 | 1 | 0 | 0 | 0 |
|  | Q1 | 1 | 1 | 1 | 0 | 1 | 1 |
|  | Median | 1 | 1 | 2 | 1 | 2 | 1 |
|  | Q3 | 2 | 2 | 3 | 2 | 4 | 2 |
|  | Max | 3 | 6 | 4 | 7 | 12 | 12 |
|  | % aligned | 20.0% | 13.3% | 47.1% | 9.9% | 37.1% | 26.7% |
| C | Mean | 2.3 | 1.8 | 2.6 | - | 2.9 | 2.2 |
|  | Min | 0 | 0 | 1 | - | 1 | 0 |
|  | Q1 | 1 | 1 | 1 | - | 2 | 1 |
|  | Median | 2 | 2 | 2 | - | 3 | 2 |
|  | Q3 | 3 | 2 | 4 | - | 4 | 2 |
|  | Max | 12 | 6 | 6 | - | 6 | 12 |
|  | % aligned | 7.5% | 3.2% | 11.1% | - | 11.8% | 6.4% |
| D | Mean | 2.5 | 2.1 | 2.8 | 1.7 | 3.4 | 2.5 |
|  | Min | 0 | 0 | 0 | 0 | 0 | 0 |
|  | Q1 | 1 | 1 | 2 | 1 | 2 | 1 |
|  | Median | 2 | 2 | 2 | 1 | 3 | 2 |
|  | Q3 | 3 | 3 | 3 | 2 | 4 | 3 |
|  | Max | 12 | 12 | 24 | 10 | 12 | 24 |
|  | % aligned | 6.4% | 4.0% | 8.5% | 2.9% | 13.8% | 7.5% |
